# A Chinese herbal formula Kesuting Syrup against COVID‐19: Leveraging multidimensional computations in network pharmacology‐driven experimental and clinical trials

**DOI:** 10.1002/ctm2.1569

**Published:** 2024-02-15

**Authors:** Cheng Zhang, Tian‐Tian Sun, Ying Lv, Xing Li, Yun Ling, Ning Wang, Wen Xia, Xiaohong Fan, Yibin Feng

**Affiliations:** ^1^ School of Chinese Medicine The University of Hong Kong Hong Kong China; ^2^ National‐Local Joint Engineering Research Center for Modern Miao Herbs Innovation Technology Anshun China; ^3^ Shanghai Public Health Clinical Center Shanghai China

Dear Editor,

The 2019 coronavirus disease (COVID‐19) outbreak has spread across the globe. Variants of the pathogen, severe acute respiratory syndrome coronavirus 2 (SARS‐CoV‐2), have been continually developing. COVID‐19 threatens people's health and hinders economic development.^1^ Using drug repurposing, Kesuting Syrup as a registered herbal formula in China can potentially treat COVID‐19. Rutin is a key compound of Kesuting Syrup for COVID‐19 therapy by regulating IFN‐γ. Multidimensional network pharmacology‐driven clinical study may be a promising method for drug development.

Kesuting Syrup, a Chinese Medicine formula, has been registered by National Medical Products Administration in China since 2002 (National registered number: Z20025238). It treated upper respiratory symptoms satisfactorily, such as sore throat and cough. The drug quality and intake safety are guaranteed since it has been used for over 20 years. The Kesuting Syrup is composed of nine herbs, including *Reineckea carnea* (JI‐Xiang‐Cao), *Solomonseal rhizome* (Huang‐Jing), *Disporum cantoniense* (Bai‐Wei‐Shen), *Platycodonis radix* (Jie‐Geng), *Saxifraga stolonifera* (Hu‐Er‐Cao in Chinese), *Eriobotryae folium* (Pi‐Pa‐Ye), *Ephedrae herba* (Ma‐Huang), *Mori Cortex* (Sang‐Bai‐Pi) and *Papaveris pericarpium* (Ying‐Su‐Ke). All the intake doses of each herb in Kesuting Syrup are in the safety range as documented in the Chinese Pharmacopoeia. Based on the long‐term clinical efficacy of Keketing Syrup, it has obvious therapeutic effect on the syndrome of cold‐damp epidemic virus invading lung due to coronavirus pneumonia in mouse model.^2^ Notably, we have also clinically validated that Kesuting Syrup may effectively treat the symptoms of COVID‐19 infected by SARS‐CoV‐2 Omicron variant.^3^ Now, the primary epidemic strain in China is also the Omicron variant.^4^, ^5^ The symptoms mainly include sore throat and cough.^6^, ^7^ Since the main symptom of COVID‐19 is cough, Keketing Syrup may be a promising drug for COVID‐19. In this study, we measured the antivirus effect of Kesuting Syrup in the in vitro study. A549 cells were infected by the HCoV‐229E virus followed by measuring the virus activity by RT‐PCR. Twenty individuals with COVID‐19 who were infected with the original strain of SARS (from the First Affiliated Hospital of Nanchang University) and 200 cases with the Omicron variant (from the Shanghai Public Health Clinical Center) were recruited from April 2020 to August 2022. In addition, we conducted a bioinformatics analysis led by network pharmacology to explore the potential therapeutic mechanism of Kesuting Syrup on COVID‐19 (Supplementary Material 1, Ingredient targets). The flowchart of this study is shown in Figure 1A. The main analytical methods are network pharmacology with multidimensional computations, molecular docking and single‐cell analysis (See Figures 2, 3, 4, 5).

**FIGURE 1:**
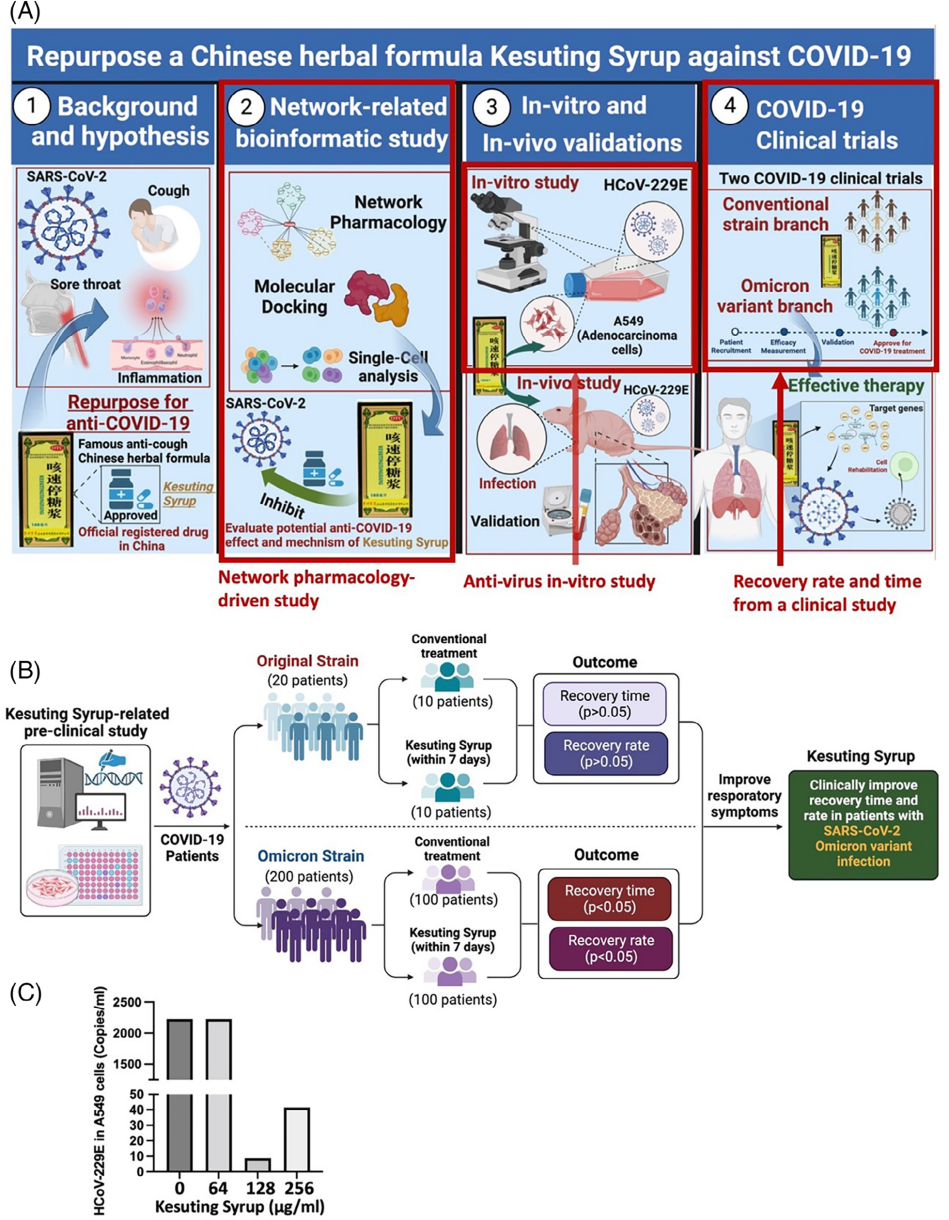
Flow chat of the study and results of in vitro virus load. (A) The highlighted areas with red squares are the contents involved in this study. The whole figure is the comprehensive strategy for repurposing Kesuting Syrup against COVID‐19. (B) The flow chat and main results for the clinical study, showing that Kesuting Syrup can clinically improve recovery time and rate in patients with SARS‐CoV‐2 mild Omicron variant infection (C) HCoV‐229E virus load in A549 cells, suggesting that Kesuting Syrup with a dose of 128 μg/mL has the most obvious antivirus effect.

**FIGURE 2:**
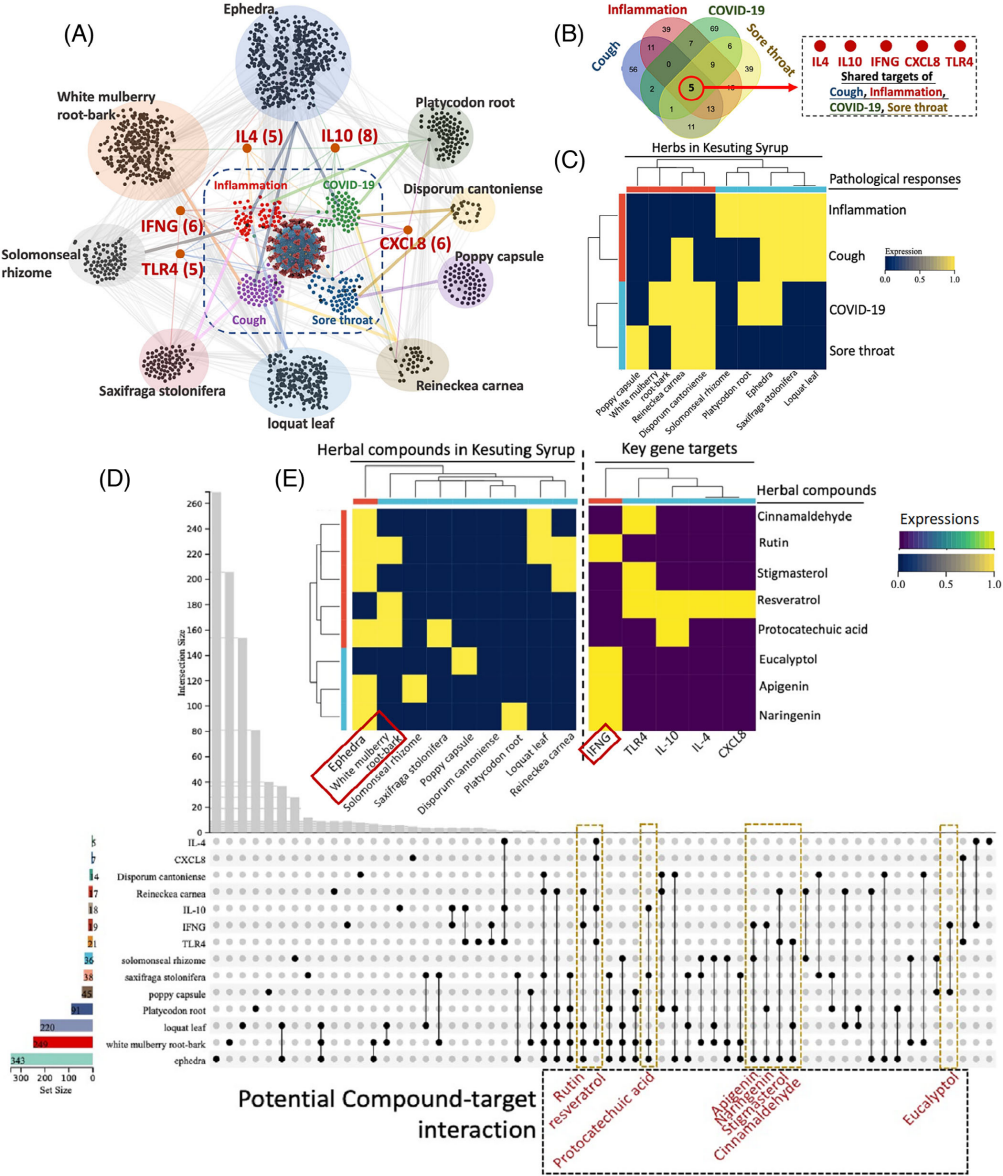
Results of network pharmacology of Kesuting Syrup against COVID‐19. (A) Network of the relationship between compounds/targets of Kesuting Syrup and COVID‐19‐related complications. (B) Venn plot sharing the shared targets of inflammation, COVID‐19, sore throat and cough. (C) The relationship between COVID‐19 symptoms and herbs in Kesuting Syrup. (D) UpsetR plot using R for show the shared targets between main targets regulated may key compounds in Kesuting Syrup (details are shown in Supplementary Material 1). (E) The relationship among active compounds in Kesuting Syrup and their targets.

**FIGURE 3:**
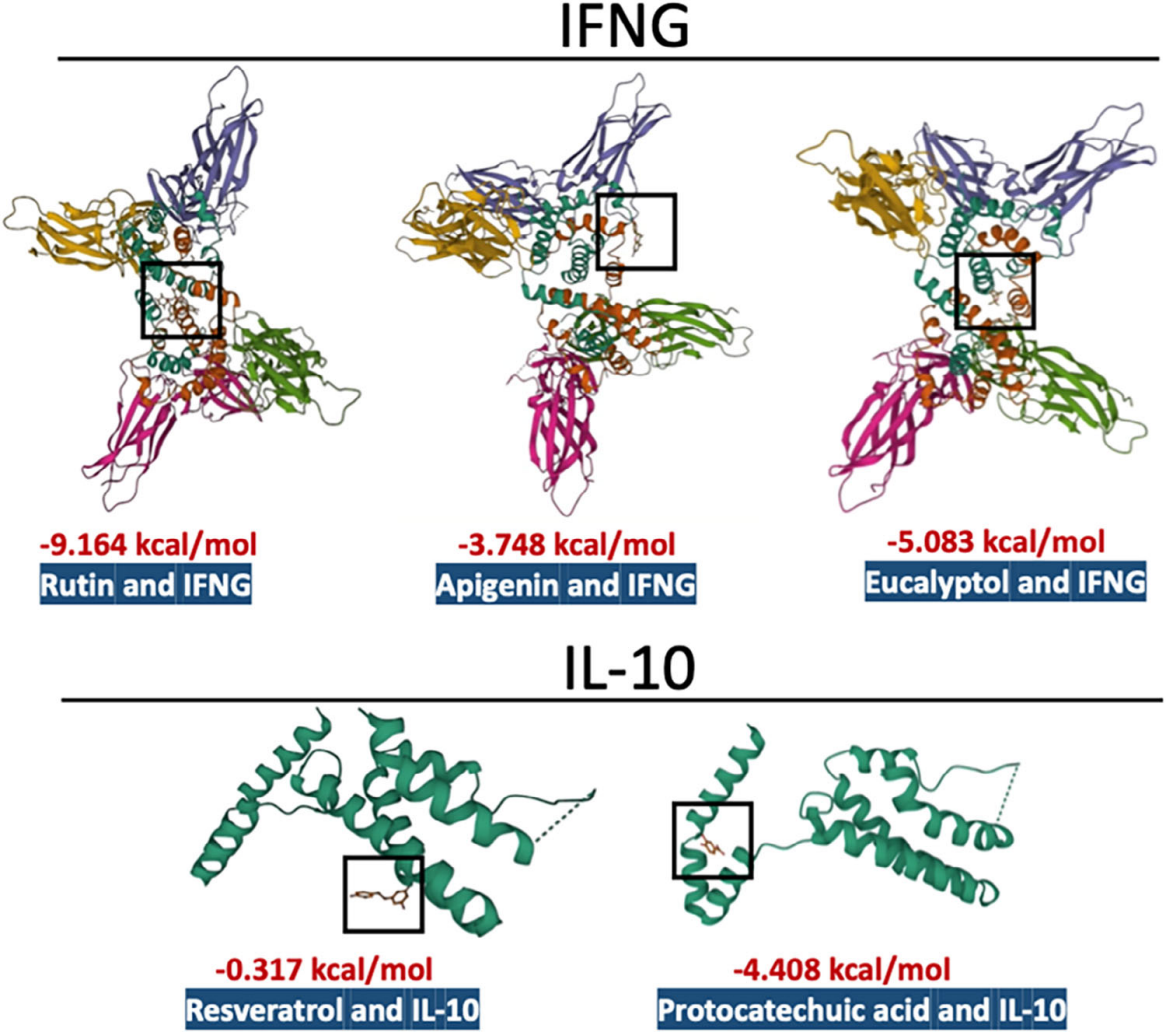
Molecular docking showing the binding affinity between key targets and proteins. The affinity of the contributive compounds (Rutin, Apigenin, Eucalyptol, Resveratrol and Protocatechuic acid) with their interactive targets, showing that the combination of Rutin and IFNG has the highest binding affinity.

**FIGURE 4:**
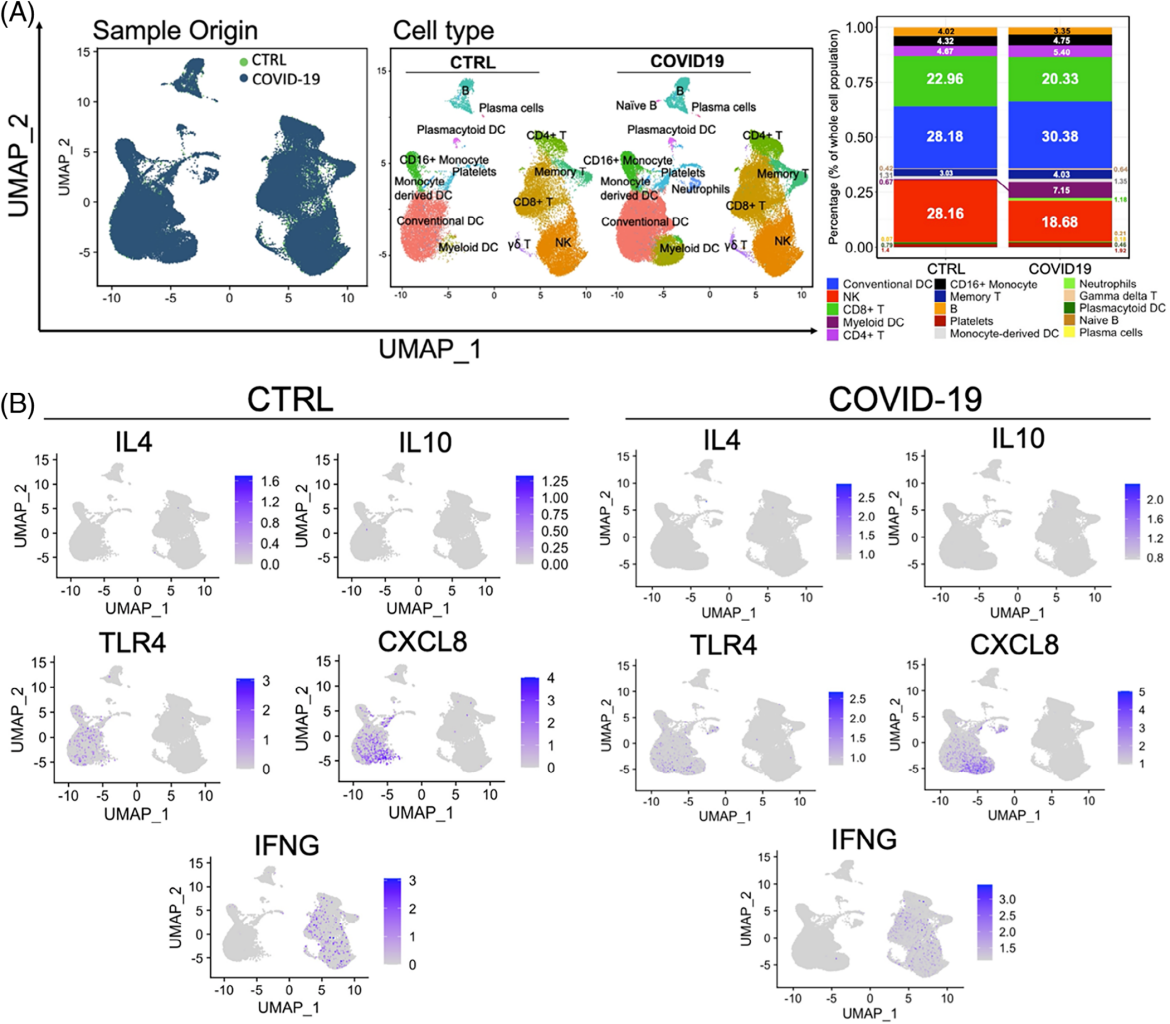
Single‐cell analysis from PBMCs between control and COVID‐19 patients (data were retrieved from NCBI‐GEO). (A) Cell type profiles from PBMCs in health or COVID‐19 patients. (B) The distribution of key modulated targets (IL4, IL‐10, TLR4, CXCL8, IFNG) by Kesuting Syrup, suggesting the distribution (not the expression) of IL4, IL‐10, TLR4, CXCL8 and IFNG between healthy individuals and COVID‐19 patients may be nonsignificant.

**FIGURE 5:**
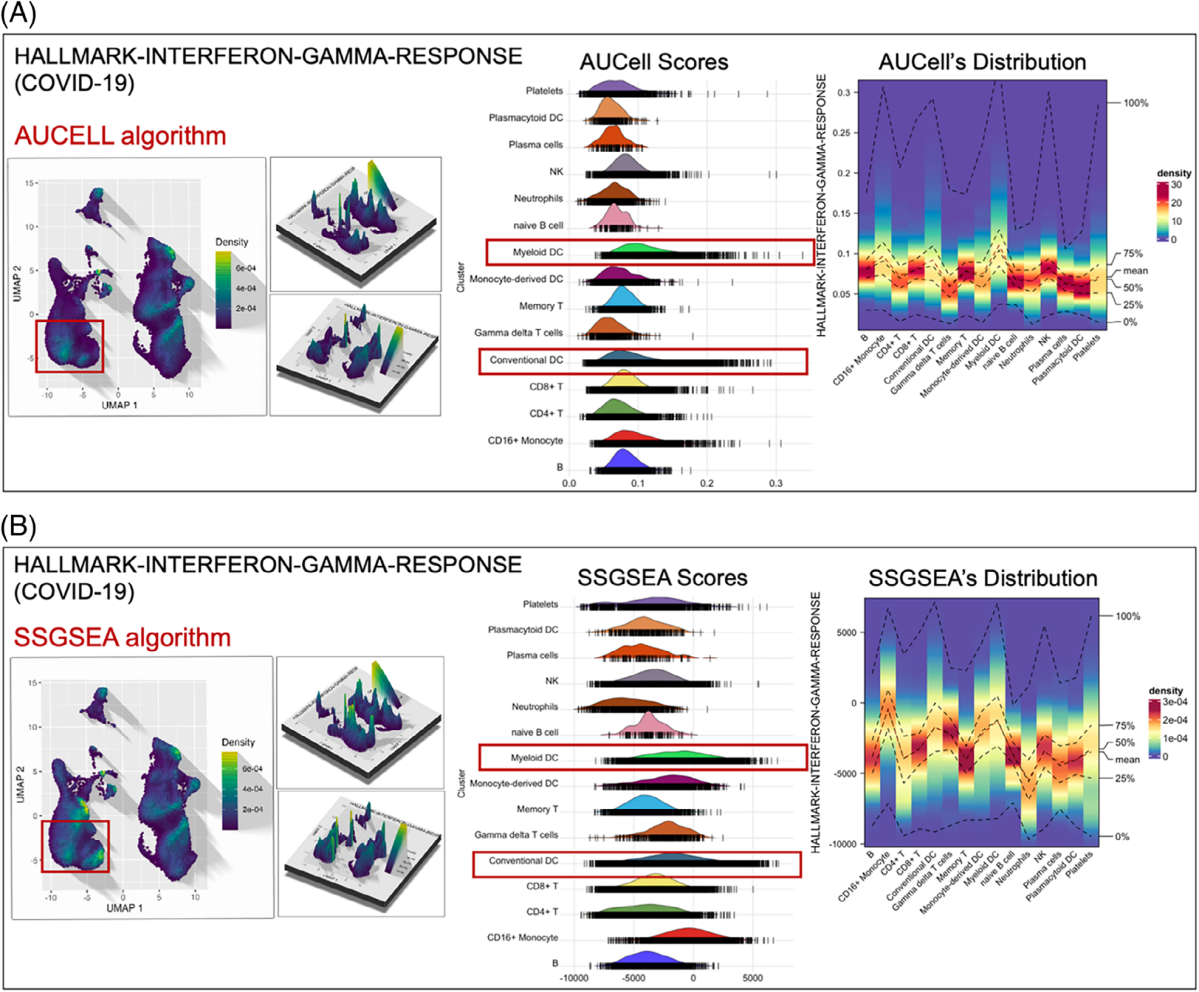
The enrichment expression of pathway ‘Interferon‐gamma‐response’ in various cell types from PBMC, suggesting that the high expression of targets involved in ‘Interferon‐gamma‐response’ are in the dendritic cells (conventional DC and myeloid DC, highlighted in red), which is responsible for generating IFN‐gamma.

For the baseline characteristics, we recruited 20 individuals with COVID‐19 who were infected with the original strain of SARS (from the First Affiliated Hospital of Nanchang University) and 200 cases with Omicron variant (Shanghai Public Health Clinical Center) from April 2020 to August 2022 (Supplementary Material 2, Tables S1 and S2). Participants were distributed to either Kesuting Syrup or Control group randomly. For the original strain, it has a Kesuting Syrup (*n* = 10) and a control branch (*n* = 10). For the Omicron strain, it has a Kesuting Syrup (*n* = 100) and a control branch (*n* = 100). The control group from the original strain is treated with the conventional therapy mentioned in the *Diagnosis and Treatment Protocol for Novel Coronavirus Pneumonia (hereinafter referred to as ‘National guideline for COVID‐19 management’)* (5th). However, for the control group from Omicron strain, the patients were treated with Lianhua Qingwen Granule as a conventional treatment. The Ethics Committee of The First Affiliated Hospital of Nanchang University authorised this study (Approval No.: 2020‐LLS‐001 for the original strain) and Shanghai Public Health Clinical Center (Approval No.: 2022‐E050‐02 for the Omicron strain) before the enrolment of subjects into the study. The diagnostic criteria are referred to the diagnostic criteria for mild COVID‐19 given in the *National guideline for COVID‐19 management*.

Regarding the therapy, for the original strain branch, the conventional therapy given in the *National guideline for COVID‐19 management (Version 5)* was combined with Kesuting Syrup (Guizhou Bailing Group Pharmaceutical Co., Ltd., Approval No.: GYZZ Z20025238) in the experimental group (100 mL per bottle, taken orally, three times a day, 20 mL each time). The conventional treatment was used in the control group. The treatment lasted 7 days (during the treatment, if the subject's cough symptoms disappeared, the Kesuting Syrup administration could be stopped at any time within the first 7 days, but the conventional therapy continued). The manuscript shared the published baseline data of the same patient population.^3^ For the Omicron branch, the conventional therapy given in the *National guideline for COVID‐19 management* (*Version 9*) was combined with Kesuting Syrup (Guizhou Bailing Group Pharmaceutical Co., Ltd., Approval No.: GYZZ Z20025238) in the experimental group (100 mL per bottle, taken orally, three times a day, 20 mL each time). The Lianhua Qingwen Granule (Beijing Yiling Pharmaceutical Co., Ltd., Approval No.: GYZZ Z20100040) was used in the control group (6 g per sachet, taken orally, three times a day, one sachet each time).^3^ As the same in the original strain branch, in the treatment period, if cough symptoms disappeared, the Kesuting Syrup therapy could be stopped at any time within the first 7 days, but the conventional therapy will continue. The clinical criteria, including inclusion and exclusion criteria, are shown in Supplementary Material 2, Methods.

Regarding the results, for the original branch in the clinical trial, there was no statistical difference between Kesuting and the control branch in terms of recovery time and recovery rate (negative within 7 days after infection) (*p* > .05). However, an obvious distinction was detected in control and treatment groups (*p* < .05) (Supplementary Material 2, Tables S3 and S4). In addition, for both the original and Omicron strain, there was no statistical difference between the Kesuting Syrup group and the control group in terms of negative conversion day (*p* > .05) (Supplementary Material 2, Tables S5 and S6). For the in vitro study, Kesuting Syrup can significantly reduce the expression of the viral nucleic acid of HCoV‐229E in A549 cells (*p* < .05). For the network result, the most regulator compounds in Kesuting Syrup for the management of COVID‐19, inflammation, sore throat and cough may include Rutin, Resveratrol, Protocatechuic acid, Apigenin, Naringenin, Stigmasterol, Chinnamaldehyde and Eucalyptol. The high‐impact targets include IL10, IL4, IFNG, CXCL8 and TLR4. In Supplementary Material 2, a detailed elaboration is given for discussing the Chinese Medicine theory and therapeutic mechanism behind Kesuting Syrup's action against COVID‐19.

In conclusion, Kesuting Syrup can clinically improve recovery time and rate in patients with SARS‐CoV‐2 mild Omicron variant infection. Additionally, Kesuting Syrup can significantly reduce the expression of viral nucleic acid. Rutin may be responsible for the anti‐SARS‐CoV‐2 effect by targeting IFNG, which is mainly from Ephedra and White mulberry root‐bark in the Kesuting Syrup. By modulating IFNG, Rutin could enhance the body's primary immune response against SARS‐CoV‐2. This action might result in more effective containment of the virus's replication and dissemination, contributing to a quicker recovery from COVID‐19. Moreover, the symptomatic improvement of Kesuting Syrup, while it may not significantly influence viral replication, can be an adjunct to standard care in managing patient comfort and may indirectly impact recovery metrics of COVID‐19.

## AUTHOR CONTRIBUTIONS

YF and XHF conceived and designed the study. The paper was mainly drafted by CZ and TTS. Data were collected and analysed by all the authors. The final version of this paper was approved by all the authors.

## CONFLICT OF INTEREST STATEMENT

The authors declare that they have no conflict of interest.

## ETHICS STATEMENT

The Ethics Committee of The First Affiliated Hospital of Nanchang University authorised this study (Approval No.: 2020‐LLS‐001 for the original strain) and Shanghai Public Health Clinical Center (Approval No.: 2022‐E050‐02 for the Omicron strain).

## Supporting information

Supporting Information

Supporting Information

## Data Availability

The data that support the findings of this study are available from the corresponding author upon reasonable request.
