# Supplementary material for: A Chinese herbal formula Kesuting Syrup against COVID‐19: Leveraging multidimensional computations in network pharmacology‐driven experimental and clinical trials
Source: Clin Transl Med. 2024 Feb 15;14(2):e1569. doi: 10.1002/ctm2.1569 (PMC10867594; doi:10.1002/ctm2.1569)
Supplement: Supplementary file 1 — Supporting Information [file CTM2-14-e1569-s001.docx]

**Supplementary Material 2:**

**Letter-to-Editor**

A Chinese herbal formula Kesuting Syrup against COVID-19: leveraging multidimensional computations in network pharmacology-driven experimental and clinical trials

(1. School of Chinese Medicine, The University of Hong Kong, Hong Kong, China; 2. [National-Local Joint Engineering Research Center](https://www.baidu.com/s?ie=utf-8&f=8&rsv_bp=1&tn=62095104_17_oem_dg&wd=National-Local%20Joint%20Engineering%20Research%20Center&oq=%25E5%259B%25BD%25E5%25AE%25B6%25E5%259C%25B0%25E6%2596%25B9%25E8%2581%2594%25E5%2590%2588%25E5%25B7%25A5%25E7%25A8%258B%25E7) for Modern Miao Herbs Innovation Technology, Anshun 561000, China; 3. Shanghai Public Health Clinical Center, Shanghai 201508, China;)

*Co-corresponding authors: Yibin FENG and Xiaohong FAN

Cheng ZHANG^1#^, Tian-tian SUN^2#^, Ying LV^3^, Xing LI^2^, Yun LING^3^, Ning WANG^1^, Wen XIA^2^, Xiaohong FAN^3*^, Yibin FENG^1*^

**1. Methods:**

**Inclusion criteria**

**For the original strain branch**

(1) mild or moderate COVID-19 infection; (2) patients with a cough score > 1 [Guidelines for Diagnosis and Treatment of Cough (2015) ^1^; (3) hospitalized patients aged 18 to 75 (inclusive), regardless of gender; (4) subjects with no plan for pregnancy, sperm donation or egg donation in the past six months, willing to take effective contraceptive measures from the first administration of the investigational drug until three months after the last administration; (5) patients willing to sign the informed consent form (ICF).

**For the Omicron branch**

(1) mild infection with SARS-CoV-2 Omicron variant; (2) patients with a cough score > 1 [Guidelines for Diagnosis and Treatment of Cough (2015) ^1^; (3) hospitalized patients aged 18 to 75 (inclusive), regardless of gender; (4) subjects with no plan for pregnancy, sperm donation or egg donation in the past six months, willing to take effective contraceptive measures from the first administration of the investigational drug until three months after the last administration; (5) patients willing to sign the informed consent form (ICF).

**Exclusion criteria**

**For the Original strain branch**

(1) Patients with moderate, severe or critical COVID-19, or COVID-19 patients requiring mechanical ventilation; (2) Patients are estimated to die within 48 hours; (3) Patients with asthma attack, suppurative tonsillitis, acute and chronic bronchitis, sinusitis, otitis media and other respiratory diseases that affect clinical trial evaluation; Patients with basic lung diseases such as severe pulmonary interstitial disease, bronchiectasis and obstructive pulmonary disease confirmed by chest CT. (4) Patients with respiratory tract infection caused by primary immunodeficiency disease, acquired immunodeficiency syndrome, congenital respiratory malformation, congenital heart disease, gastroesophageal reflux disease, pulmonary dysplasia and other basic diseases. (5) Patients who had or have chronic or serious diseases that may affect the admission into the trial or the outcome of the study; (6) Those who may be uncooperative due to poor mental health, or those suffering from mental illness or incapable of controlling themselves or expressing themselves clearly; (7) Patients with poor hypertension control (min. pressure ≥ 110 mmHg or max. pressure ≥ 180 mmHg); (8) Patients with alanine transaminase (ALT) or aspartate aminotransferase (AST) ≥ 1.5 times the upper limit of normal (ULN), or serum creatinine (Scr) > ULN; (9) Those with a history of specific allergies such as asthma, measles and eczema, or those allergic to two or more drugs or foods such as milk and pollen, or those allergic to the ingredients of Kesuting Syrup; (10) Those with a history of drug abuse or dependence within 6 months before randomization; (11) Pregnant or lactating women; (12) Patients who participated in or are participating in clinical trials of other drugs within 3 months before screening; (13) Other circumstances judged by the investigator to be unsuitable to participate in this clinical trial.

**For the Omicron branch**

(1) Patients infected by other COVID-19 variants or patients with moderate, severe or critical COVID-19, or COVID-19 patients requiring mechanical ventilation; (2) Patients with other respiratory diseases that could affect the clinical trial assessment, or patients with serious underlying lung diseases; (3) Patients with respiratory tract infection caused by underlying diseases such as immune deficiency and congenital respiratory tract abnormality; (4) Patients who had or have chronic or serious diseases that may affect the admission into the trial or the outcome of the study; (5) Those who may be uncooperative due to poor mental health, or those suffering from mental illness or incapable of controlling themselves or expressing themselves clearly; (6) Diabetes patients; (7) Patients with poor hypertension control (min. pressure ≥ 110 mmHg or max. pressure ≥ 180 mmHg); (8) Patients with alanine transaminase (ALT) or aspartate aminotransferase (AST) ≥ 1.5 times the upper limit of normal (ULN), or serum creatinine (Scr) > ULN; (9) Those with a history of specific allergies such as asthma, measles and eczema, or those allergic to two or more drugs or foods such as milk and pollen, or those allergic to the ingredients of Kesuting Syrup or Lianhua Qingwen Granule; (10) Those with a history of drug abuse or dependence within 6 months before randomization; (11) Those who have used any traditional Chinese medicine or western medicine for relieving cough and resolving phlegm within 24 hours before randomization; (12) Pregnant or lactating women; (13) Patients who participated in or are participating in clinical trials of other drugs within 3 months before screening; (14) Other circumstances judged by the investigator to be unsuitable to participate in this clinical trial.

**PCR-related Virus nucleic acid test for HCoV-229E load in A549 cells**

A549 cells were purchased from BNCC (Beijing, China). A549 cells were treated with or without Kesuting Syrup, ranging from 32μg/ml to 256μg/ml. The virus nucleic copies in the control group is 2.23*10^7^ copies/ml. For the PCR-related test, DEPC-H2O was used as the negative control, and the positive control was diluted in 10-, 100-, and 1000-fold gradients. For PCR amplification, the CT value is set to 40, and the single-point fluorescence detection is at 60 degrees.

**Measurement of the recovery time and rate in the clinical trial**

Recovery time: the patient's body temperature returned to 37℃ for more than 3 days. The respiratory symptoms improved significantly, and the SARS-CoV-2 nucleic acid test was negative for two consecutive times. For the recovery rate, it is the number of recovered cases (negative with 7 days after infection) divided by total cases and multiplied by 100%

**Calculation of sample size**

Since the available patients from the original SARS-CoV-2 branch is very limited, the final included cases in either Kesuting treatment and control group is 10 patients in each group. While, for the sample size calculation for this clinical study (Omicron branch), we estimated that around 100 individuals would be involved in either the treatment and control. Therefore, 200 individuals (100*2 groups) will be included.

Here are the calculation details.

We estimated that the Chinese medicine groups have a 20% improvement rate when compared with the placebo group. Level of significance = 5%, Power = 80%, Type of test = two-sided. The sample size is calculated by a well-known equation as follows ^2^.

Equation (sample size in each group):

N = [(Zα/2 + Zβ)^2^ × {(p1 (1-p1) + (p2 (1-p2))}] / (p1 - p2)^2^

where,

n = Sample size required in each group,

p1 = Percent changes of subjects improved by Chinese medicine treatment = 0.2

p2 = Percent changes of subjects improved by placebo treatment = 0.05,

p1-p2 = clinically significant difference =0.15

Zα/2: This depends on the level of significance for 5% is 1.96

Zβ: This depends on power for 80% is 0.84

Based on the above formula the sample size required per group is [7.84*(0.16+0.0475)] / (0.15)^2^=72.3. If the drop-out rate is 30%, the number is 72.3+(72.3*0.3) = 93.99 (approximately 100) subjects in each group. Hence, the total sample size of the 2 groups in the Omicron branch was 100*2 groups=200 subjects.

**Network pharmacology**

Pharmacological actions can be thought of as a "key" to fit into a specific "lock". Rather than enumerating case studies separately, drug actions elaborated through network pharmacology can provide more in-depth information regarding the pharmacological actions of Chinese medicine and its targets.

The collected data from the databases are imported into "Cytoscape" for network analysis following a large-scale screening of compounds and targets from the database HERB (http://herb.ac.cn). Chinese medicine, pure chemicals, and targets are represented as nodes in the network. Simultaneously, interactions are represented by the nodes' edges, which connect nodes directly. Nodes with more centripetal position accounts and shared edges perform better than those with fewer. A statistical plug-in called "NetworkAnalyzer" that is coupled to Cytoscape has performed network analysis to provide a deeper understanding by calculating the correlation degree for each node. The network displays the nine herbs together with the linked compounds and targets. The UpsetR in R is used to visualize overlapped targets among genes and active compounds.

**Molecular Docking**

Using AutodockVina (version 1.2.2), the binding affinities and mechanisms of interaction between therapeutic candidates and their targets were examined. From PubChem Compound (https://pubchem.ncbi.nlm.nih.gov), the molecular structures were obtained. The protein as well as the molecular data were converted into PDBQT format followed by docking analysis. The grid box was positioned in the middle to allow for free molecular movement and to cover the domain of each protein. Grid point distance was set at 0.05 nm. Autodock Vina (http://autodock.scripps.edu/) was used to conduct molecular docking investigations.

**Single-cell analysis**

The single-cell dataset (GSE165080) was retrieved from NCBI-GEO database (PBMC from control or COVID-19 patients). The Seurat function (version 4.1.0) in R was adopted for normalization (DESeq2 function), dimensional reduction and, cell population clustering. PanglaoDB-related gene annotation was used for labelling the specific cell population. The irGSEA package was used for the single-cell mechanism study. The data visualization was mainly performed by ggplot2.

**Statistical method**

R software was used for statistical analysis. Skewed continuous variables were presented as mean (25^th^ percentile, 75^th^ percentile) and comparation between groups were analyzed by Wilcoxon rank sum test. The categorical variables were shown as number of subjects (percent) and compared between groups using χ^２^ test or fisher exact test as appropriate. P＜0.05 was considered as the statistical significance.

**2. Results**

**Outcome measures**

**Network pharmacology**

For the network result, we screened the contributive compounds and targets of Kesuting Syrup for the suppression of COVID-19, inflammation, Sore throat, and Cough. As a result, the high-influence compounds are Rutin, Resveratrol, Protocatechuic acid, Apigenin, Naringenin, Stigmasterol, Chinnamaldehyde, and Eucalyptol. For the identification of the regulatory targets, we first screened the shared targets of COVID-19, inflammation, Sore throat, and Cough, indicating that IL10, IL4, IFNG, CXCL8, and TLR4 are the most high-impact ones. Coincidently, these 5 targets are also the high-degree targets in the network (ranked within the Top 10), indicating that the inhibitory effect of Kesuting Syrup may be related to the regulation of IL10, IL4, IFNG, CXCL8, and TLR4 (Figure 2)

**Molecular docking**

For the molecular docking, it is used to evaluate the affinity of the candidate drugs for their targets. Using Autodock, the binding affinity for each interaction was obtained (Figure 3). Each drug candidate will be tried to bind to its protein targets via hydrogen bonds and electrostatic interactions. In addition, the results indicated that hydrophobic pockets of targets were occupied successfully by the Rutin, Resveratrol, Protocatechuic acid, Apigenin, Naringenin, Stigmasterol, Chinnamaldehyde, and Eucalyptol, showing that Rutin and IFNG have the highest affinity (-9.164kcal/mol)

**Single-cell analysis**

Single-cell analysis from PBMCs between control and COVID-19 patients (data were retrieved from NCBI-GEO) were conducted. The cell type profiles, including the proportion of each cell type, were visualized by UMAP (Figure 4A). In addition, the distribution of key modulated targets (IL4, IL-10, TLR4, CXCL8, IFNG) regulated by Kesuting Syrup was shown in Figure 4B. It suggested that the distribution of IL4, IL-10, TLR4, CXCL8, and IFNG between healthy individuals and COVID-19 patients may be non-significant. However, the similar target distribution is not equal to the similar gene and functional pathway expression in cell types. Since IFNG is the main target of Kesuting Syrup as demonstrated by network pharmacology, we further conducted the pathway enrichment analysis targeting the pathway of “Interferon-gamma response” in various cell types using AUCELL algorithm and SSGSEA algorithm (Figure 5). It suggested that the high expression of targets involved in “Interferon-gamma-response” are in the dendritic cells (Conventional DC and Myeloid DC), which is may responsible for generating IFNG.

**Inhibitory effect of Kesuting Syrup on inhibition of HCoV-229E**

In Figure 1B, we measured the HCoV-229E virus load in A549 cells. The results indicated that Keusuting Syrup with a dose of 128μg/ml has the most obvious anti-virus effect.

**Comparison of baseline characteristics between two groups**

**For the original branch**

For the groups of the original branch, 10 men and 10 women with mild [symptom](http://dict.youdao.com/w/symptom/#keyfrom=E2Ctranslation)s were included (4 men and 6 women). There was no statistical difference between the experimental and control group in baseline characteristics (*P*>0.05) (Table 1).

**For the Omicron branch**

In the groups of the Omicron branch, 109 men and 91 women with mild [symptom](http://dict.youdao.com/w/symptom/#keyfrom=E2Ctranslation)s were included. There was no statistical difference between the experimental and control group in baseline characteristics (P>0.05) (Table 2).

**2.6 Comparison of** **recovery rate and time after treatment**

For the recovery time, those patients’ body temperature returned to 37℃ for more than 3 days has been included. Also, the respiratory symptoms should be improved, and the SARS-CoV-2 nucleic acid test was negative for two consecutive times.

**For the Original branch**

For the original branch, it indicated that there was no statistical difference between the Kesuting Syrup group and the control group in terms of recovery time and rate (p>0.05). The recovery rate is 80% (Kesuting Syrup group) and 30% (vehicle control group), respectively. The recovery time is 3.44±1.77 days (Kesuting Syrup group) and 4.85±2.19 days (control group), respectively (Table 3). It suggested that Kesuting Syrup cannot improve the recovery time and rate in patients with original SARS-CoV-2 infection.

**For the Omicron branch**

The recovery time and rate in patients infected by the Omicron strain, there is a significant difference in the control and treatment groups (p<0.05). The recovery rate is 59% (Kesuting Syrup group) and 22% (control group), respectively. The recovery time is 8.53±3.37 days (Kesuting Syrup group) and 10.74±2.58 days (control group), respectively. (Table 4). It suggested that Kesuting Syrup can significantly improve the recovery time and rate in patients with SARS-CoV-2 Omicron variant infection.

**Comparison of** **negative conversion day**

**For the Original branch**

For the original branch, it suggested that there was no statistical difference between the Kesuting Syrup group and the control group in terms of negative conversion day (p>0.05). The negative conversion day is 2.92 ±1.8 days (Kesuting Syrup group) and 4.21 ±2.55 days (control group), respectively (Table 5). It suggested that Kesuting Syrup cannot reduce the period of negative conversion day in patients with original SARS-CoV-2 infection.

**For the Omicron branch**

For the Omicron branch, it suggested that there was no statistical difference between the Kesuting Syrup group and the control group in terms of negative conversion day (p>0.05). The negative conversion day is 5.66 ±2.99 days (Kesuting Syrup group) and 5.11 ±3.62 days (control group), respectively (Table 6). It suggested that Kesuting Syrup cannot decrease the period of negative conversion day in patients with infection of the SARS-CoV-2 Omicron variant.

**3. Discussion**

COVID-19, officially named by the WHO in November 2021, is characterized by high transmissibility and fast transmission, with the Omicron variant exhibiting particularly strong concealment^3^. It was reported that the main symptoms of COVID-19 include cough, sore throat, and nasal congestion.^4^ The Chinese medicine syndromes were dominated by wind-heat invading lung and spleen-lung qi deficiency. It was reported that COVID-19 was considered to be a turbid toxin-induced epidemic disease in traditional Chinese medicine theory^5^. Such toxins can contaminate the human body, making it easy for blood stasis and phlegm to occur. They believed that spleen-lung qi deficiency is the root cause of the disease, but the onset of the disease is triggered by turbid toxin combined with blood stasis and phlegm which can aggravate the disease when blocking meridians.

Kesuting Syrup, with its Chinese medicine roots in invigorating qi and nourishing yin, moistening the lung, relieving cough, tonifying the stomach, and promoting fluid, has been investigated for its potential in treating COVID-19^6^. A balance between Yin (cooling, moistening) and Yang (warming, activating) elements is crucial for the treatment of infectious diseases, including COVID-19. In Kesuting Syrup, certain herbs contribute to this balancing act. For instance, herbs like Eriobotryae folium and Mori Cortex, known for their cooling properties, could be seen as contributing to the Yin aspect, helping to reduce inflammation and heat symptoms associated with COVID-19. On the other hand, herbs like Ephedrae herba, which has warming properties, contribute to the Yang aspect, potentially aiding in improving respiratory function and energy levels. The synergistic effect of these herbs may contribute to maintaining the balance of Yin and Yang against COVID-19. For the potential therapeutic mechanism of Chinese medicines in Kesuting Syrup, it may include anti-inflammatory and pain-relieving properties (Reineckea carnea), nourishment for lungs and stomach (Solomonseal rhizome), immune system enhancement (Disporum cantoniense), respiratory relief by treating cough and clearing phlegm (Platycodonis radix, Eriobotryae folium), reduction in systemic inflammation (Saxifraga stolonifera), bronchodilation and decongestion (Ephedrae herba), lung health improvement and edema reduction (Mori Cortex), and symptomatic relief with sedative and cough-suppressing effects (Papaveris pericarpium). Each herb's traditional use and known pharmacological properties contribute to the formula's overall effectiveness in treating COVID-19 symptoms.

As discussed above, our previous animal experiment results showed Kesuting Syrup could significantly reduce the inflammatory damage of lung tissue in mice. It can lower the expression level of IL-6, IL-10, TNF- α and IFNG, and improve pathological manifestations such as alveolar septal edema and inflammatory cell infiltration.^7^. In this study, the antiviral effect of Kesuting Syrup was further validated through in-vitro studies, which forms the basis for network pharmacology and clinical studies positioning Kesuting Syrup as a potential COVID-19 treatment. Kesuting Syrup was evaluated in a case-control clinical study on patients infected with either the SARS-CoV-2 original strain or the Omicron variant. While there was no significant difference in negative conversion day between the Kesuting Syrup and control groups for both strains, a notable improvement was observed in recovery time and rate in patients with the Omicron variant, highlighting the specific efficacy of Kesuting Syrup for this strain.

Network pharmacology analysis identified IL10, IL4, IFNG, CXCL8, and TLR4 as the most contributive targets regulated by Kesuting Syrup, with IL-10 and IFNG being particularly effective in reducing cough symptoms. Firstly, the regulation of IL-10, an anti-inflammatory cytokine, by Kesuting Syrup may be crucial in reducing the severity of the inflammatory responses seen in patients with COVID-19. This modulation is particularly important in mitigating cytokine storms, which are a critical factor in severe COVID-19 cases, leading to reduced lung tissue damage and improved patient outcomes ^8^. Furthermore, IL-4 plays a significant role in stimulating the production of antibodies ^9^. The involvement of Kesuting Syrup in regulating IL-4 could enhance the body's capacity to produce specific antibodies against SARS-CoV-2, aiding in neutralization of the virus and facilitating recovery. The targeting of CXCL8, a chemokine involved in the recruitment of immune cells to infection sites, is also significant ^10^. Kesuting Syrup's effect on CXCL8 may help the recruitment and activation of immune cells, thus preventing excessive inflammation and the associated tissue damage. Additionally, the regulation of Toll-like receptor 4 (TLR4) by Kesuting Syrup might enhance the body's ability to recognize and respond to SARS-CoV-2 more efficiently. The TLR4 related improvement of innate immune response is crucial for an effective defense against the virus ^11^. Interferon-gamma (IFNG) is another key player in antiviral defense, activating macrophages and enhancing antigen presentation. The modulation of IFNG by Kesuting Syrup may amplify the initial immune response against SARS-CoV-2, potentially curbing viral replication and spread within the host. The pathway of “Interferon-gamma (IFNG) response” was highly enriched in the dendritic cells (Conventional DC and Myeloid DC). Since dendritic cells are one of the main producers of IFNG, Kesuting Syrup may regulate dendritic cells-related production of IFNG in the treatment of COVID-19. Molecular docking results revealed that rutin, a compound highly present in Ephedra and White mulberry root-bark in Kesuting Syrup, has a high binding affinity with IFNG. This suggests that these components of Kesuting Syrup might be responsible for its antiviral effect against SARS-CoV-2 by targeting IFNG. Overall, Kesuting Syrup has been shown to clinically improve recovery time and rate in patients with mild SARS-CoV-2 Omicron variant infection and significantly reduce the expression of viral nucleic acid, with rutin potentially playing a key role in this antiviral effect. The regulation of IFNG by rutin may potentially strengthen the body's initial immune response to SARS-CoV-2. It may lead to more efficient control of viral replication and spread, aiding in faster recovery from COVID-19.

**References**

[1] Lai Kefang. Guidelines for Diagnosis and Treatment of Cough. Chinese Journal of Tuberculosis and Respiratory Diseases, 2016, 39 (05): 323-354.

[2] Sakpal, T.V., Sample size estimation in a clinical trial. Perspect Clin Res, 2010. 1(2): p. 67-9.

[3] Callaway E. Heavily mutated Omicron variant puts scientists on alert. Nature, 2021, 600 (7887) :21.

[4] Chen Bowu, Yang Mengqi, Wen Zonglin, Shen Jiaojiao, Yang Yanbing, and Gao Yueqiu. Study on TCM syndrome characteristics of patients with mild infection with SARS-CoV-2 Omicron variant in Shanghai [J]. Journal of Traditional Chinese Medicine, 2022, 63 (15): 1436-1441.

[5] Liu Xiaofa, Li Diangui, Chen Fenqiao, et al. Treatment of COVID-19 using turbid toxin theory. China Journal of Traditional Chinese Medicine and Pharmacy, 2022, 37 (08): 4503-4506.

[6] Feng Tingting, Zhang Jianfei, Lin Bing, et al. Study on the antitussive, expectorant and antiasthmatic effects of Miao herbal medicine Kesuting Capsule. Journal of Medicine and Pharmacy of Chinese Minorities, 2018, 24 (11): 55-57.

[7] Zhao Ronghua, Sun Jing, Shi Yujing, et al. Effect of intervention by the method of dispersing lung and removing phlegm on the syndrome of cold-damp epidemic virus invading lung due to coronavirus pneumonia in mouse model. Chinese Journal of Experimental Traditional Medical Formulae, 2020, 26 (11): 21-27.

[8] Azaiz, M. B., Jemaa, A. B., Sellami, W., et al. Deciphering the balance of IL-6/IL-10 cytokines in severe to critical COVID-19 patients. Immunobiology, 2022, 227(4), 152236.

[9] Lu, Q., Zhu, Z., Tan, C., Zhou, H., et al. Changes of serum IL‐10, IL‐1β, IL‐6, MCP‐1, TNF‐α, IP‐10 and IL‐4 in COVID‐19 patients. International journal of clinical practice, 2021, 75(9), e14462.

[10] Sokol, C. L., & Luster, A. D. The chemokine system in innate immunity. Cold Spring Harbor perspectives in biology, 2015, 7(5), a016303.

[11] Birra, D., Benucci, M., Landolfi, L., et al. COVID 19: a clue from innate immunity. Immunologic research, 2020, 68, 161-168.

**Tables:**

**Table 1 Comparison of Baseline Characteristics between Two Groups (original strain branch)**

|  |  | Experimental group  (n=10) | Control group  (n=10) | P value |  |
| --- | --- | --- | --- | --- | --- |
| Gender |  |  |  | 0.7249 |  |
|  | Male | 4 | 6 |  |  |
|  | Female | 6 | 4 |  |  |
| Age |  |  |  | 0.0636 |  |
|  | N (Missing) | 10 (0) | 10 (0) |  |  |
|  | Mean (SD) | 48.7(12.05) | 53.6(13.66) |  |  |
|  | Median (Q1, Q3) | 45.5(41, 55) | 58.5(42, 66) |  |  |
|  | Min, Max | 33, 69 | 34, 70 |  | |
|  |  |  |  |  | |

**Table 2 Comparison of Baseline Characteristics between Two Groups (Omicron branch, published data)**

|  |  | Experimental group  (n=100) | Control group  (n=100) | P value |
| --- | --- | --- | --- | --- |
| Gender |  |  |  | 0.155 |
|  | Male | 49 | 60 |  |
|  | Female | 51 | 40 |  |
| Age |  |  |  | 0.118 |
|  | N(Missing) | 99(1) | 100 (0) |  |
|  | Mean (SD) | 42.63 (16.32) | 38.98 (16.25) |  |
|  | Median(Q1,Q3) | 42(26.5, 56) | 37(23, 51) |  |
|  | Min, Max | 18, 71 | 18, 72 |  |

**Table 3. Comparison of recovery rate and time (original strain branch)**

|  |  | Experimental group  (n=10) | | Control group  (n=10) | P value |
| --- | --- | --- | --- | --- | --- |
| Recovery rate |  |  |  | | 0.0698 |
|  | N (Missing) | 10 (0) | 10 (0) | |  |
|  | Yes (%) | 8 (80.00) | 3 (30.00) | |  |
|  | No | 2 (20.00) | 7 (0.00) | |  |
|  |  |  |  | |  |
| Recovery time |  |  |  | | 0.2960 |
|  | N (Missing) | 8 (0) | 3(0) | |  |
|  | Mean (SD) | 3.44 (1.77) | 4.85 (2.19) | |  |
|  | Median (Q1, Q3) | 3.24 (2.20, 5.20) | 5.88 (2.33, 6.33) | |  |
|  | Min, Max | 0.78, 5.49 | 2.33, 6.33 | |  |

**Table 4. Comparison of recovery rate and time (Omicron strain branch)**

|  |  | Experimental group  (n=100) | | Control group  (n=100) | P value |
| --- | --- | --- | --- | --- | --- |
| Recovery rate |  |  |  | | ＜0.001 |
|  | N (Missing) | 100(0) | 100(0) | |  |
|  | Yes (%) | 59 (59.00) | 22 (22.00) | |  |
|  | No | 41 (41.00) | 78 (78.00) | |  |
|  |  |  |  | |  |
| Recovery time |  |  |  | | ＜0.001 |
|  | N (Missing) | 100 (0) | 100 (0) | |  |
|  | Mean (SD) | 8.53 (3.37) | 10.74 (2.58) | |  |
|  | Median (Q1, Q3) | 9 (5, 12) | 12 (12, 12) | |  |
|  | Min, Max | 2, 12 | 2, 12 | |  |

**Table 5. Comparison of negative conversion day (original strain branch)**

|  |  | Experimental group  (n=10) | | Control group  (n=10) | P value |
| --- | --- | --- | --- | --- | --- |
| Recovery time |  |  |  | | 0.4274 |
|  | N (Missing) | 5(5) | 3(7) | |  |
|  | Mean (SD) | 2.92 (1.8) | 4.21 (2.55) | |  |
|  | Median (Q1, Q3) | 2.94 (2.04, 4.24) | 4.79 (1.42, 6.42) | |  |
|  | Min, Max | 0.41, 4.94 | 1.42, 6.42 | |  |

**Table 6. Comparison of negative conversion day (Omicron strain branch)**

|  |  | Experimental group  (n=100) | | Control group  (n=100) | P value |
| --- | --- | --- | --- | --- | --- |
| Recovery time |  |  |  | | 0.111 |
|  | N (Missing) | 49 (51) | 51(49) | |  |
|  | Mean (SD) | 5.66 (2.99) | 5.11 (3.62) | |  |
|  | Median (Q1, Q3) | 6 (4, 8) | 4 (2, 8) | |  |
|  | Min, Max | 0, 13 | 0, 13 | |  |
